# Spatial Allocation Rationality Analysis of Medical Resources Based on Multi-Source Data: Case Study of Taiyuan, China

**DOI:** 10.3390/healthcare12161669

**Published:** 2024-08-21

**Authors:** Lujin Hu, Shengqi Cai

**Affiliations:** School of Geomatics and Urban Spatial Informatics, Beijing University of Civil Engineering and Architecture, Beijing 102616, China; 201804010218@stu.bucea.edu.cn

**Keywords:** rationality evaluation model, supply and demand of medical resources, spatial layout, Huff-2SFCA, Taiyuan

## Abstract

Reasonably allocating medical resources can effsectively optimize the utilization efficiency of such resources. This paper took Taiyuan City as an example and established a model to evaluate the rationality of medical resource spatial allocation, incorporating two key dimensions: the spatial layout and the supply and demand of medical resources. In terms of the spatial layout, three indexes were included: Firstly, the service coverage rates of different levels of medical institutions, based on residents’ medical orientations, were calculated using network analysis methods. Secondly, the Huff-2SFCA method was improved to calculate the accessibility of medical resources for four different modes of transportation. Then, the Health Resource Agglomeration Degree (HRAD) and Population Agglomeration Degree (PAD) were used to quantify the equity of medical resources. In terms of the supply and demand of medical resources, one index was included: the supply–demand ratio of medical resources during sudden public health events, which was calculated using the number of beds per thousand people as an indicator. These four indexes were weighted using the entropy weight method to obtain the rationality grade of medical resource spatial allocation in Taiyuan City. The study found that the rationality evaluation level of medical resource allocation in the central urban area of Taiyuan City followed a “concentrically decreasing” pattern. The rating ranged from “very reasonable” to “less reasonable”, with the area of each level expanding gradually. The areas rated within the top two categories only accounted for 19.92% of the study area, while the area rated as “less reasonable” occupied 38.73% of the total area. These results indicate that the model accounted for residents’ travel for various medical orientations and the availability of resources during public health emergencies. It considered both the spatial layout and supply and demand of medical resources, offering recommendations for the precise allocation of urban medical resources.

## 1. Introduction

As one of the most populous countries in the world, China faces significant challenges in allocating medical resources. The difficulty in accessing medical care has garnered widespread attention and concern from the government, health departments, and the public alike [1]. In April 2023, the National Health Commission of the People’s Republic of China held a press conference on the expansion and regional allocation balance of high-quality medical resources. During the conference, strengthening the rational allocation of medical resources to meet the healthcare needs of the population was proposed [2]. As geographic information technologies become more widespread, an increasing number of scholars are relying on these technologies to provide precise data support and analytical tools when studying the rationality of the spatial allocation of medical resources. Geographic Information Systems (GISs) process and visualize geospatial data, helping researchers to better understand the distribution of medical resources, identify imbalances in resource allocation within regions, and propose scientific improvement suggestions [3,4]. For example, Xu et al. [5] studied the allocation characteristics and service coverage of medical facilities in Lhasa using geospatial analysis methods such as the kernel density estimation method, Thiessen polygon method, and buffer analysis. F. Javier Otamendi et al. [6] defined and developed a set of indicators based on dynamic isochrones to assess the impact of traffic accidents on public health. Lee et al. [7] proposed a novel approach using a Geographic Information System (GIS)-based spatial analysis to assist ambulance service planners and designers in assessing and providing rational service coverage based on simulated random incidents. Yang et al. [8] improved the decay function in the 2SFCA method and assessed the spatial accessibility of medical facilities at various levels in the central urban area of Shanghai. Jin et al. [9] proposed a multiple-mode Huff-based 2SFCA method and validated it in Austin, Texas. The method showed that it more accurately reflects the accessibility disparities among various types of food stores compared to the single-mode Huff-2SFCA. Wei et al. [10] improved the greedy dropping heuristic algorithm and applied it to the site selection of designated hospitals in Anhui Province during the COVID-19 pandemic. Fadahunsi, J.T. et al. [11] proposed a cartographic model to determine the optimal locations for medical institutions and applied it in Osun State, Nigeria. Han et al. [12] proposed a method for evaluating the equity of health resource allocation from both the supply and demand sides using grids as units. Yin et al. [13] utilized indicators of natural, social, and economic factors to analyze the causes of inequality in medical services in China. Liu et al. [14] employed the Gini coefficient and a data envelopment analysis to establish a method for assessing the spatial equity and efficiency of medical resources, providing a scientific basis for promoting equitable medical services in Qinghai Province. Fu Runde et al. [15] applied the OSMNHDC method to calculate the distance costs from residential areas in Xinjiang to the nearest medical facilities and analyzed the spatial equity in the allocation of healthcare resources.

In studies on the spatial allocation of medical resources, common analytical perspectives include service area analyses of medical institutions, spatial accessibility analyses of medical resources, and spatial equity analyses of medical resources. Service area analysis evaluates the geographic areas that medical institutions can serve, assessing their coverage to help planning departments identify blind spots and optimize the layout of medical resources. Spatial accessibility analysis focuses on residents’ perspectives, assessing the ease with which residents can reach medical institutions and measuring the actual service effectiveness of medical resources [16]. This helps to identify areas with poor transportation or resource shortages, providing data support for improving medical service accessibility. Spatial equity analysis examines the distribution of medical resources across different regions and socioeconomic groups, assessing whether their allocation is fair. This helps to identify areas where medical resources are overly concentrated or insufficient, focusing on the accessibility of medical services for vulnerable groups and proposing policy recommendations to achieve equitable resource allocation [17].

Additionally, the evaluation of the supply–demand relationship in the allocation of medical resources is also an important indicator that cannot be ignored [18,19]. Existing research mainly focuses on changes in the supply–demand relationship of medical resources over long time series, optimization strategies [20], and policy recommendations [21]. The sudden outbreak of the COVID-19 pandemic in 2019 sounded the alarm, drawing attention to the issue of balancing the supply and demand of medical resources during public health emergencies [22]. Compared to predicting the supply–demand relationship during the pandemic, studying how to reasonably allocate and adjust existing medical resources during safe periods to maintain overall stability during periods of increased medical demand seems to be more universal and forward-looking.

In summary, existing studies still face the following problems: (1) The spatial allocation rationality evaluation system of medical resources is not comprehensive. It usually focuses on either the spatial layout or the supply and demand of medical resources [23], without integrating both aspects into a unified system. (2) From the perspective of resource supply and demand, there is a lack of research on the characteristics of residents’ actual medical needs during public health emergencies and the pre-allocation of resources based on existing medical resources.

To address the identified issues, a comprehensive evaluation index system for the rational allocation of medical resources is established in this study, encompassing both the spatial layout and the supply and demand of medical resources. An innovative evaluation indicators system is proposed across two dimensions: (1) From the perspective of the supply and demand of medical resources, this study introduces Dianping data from the COVID-19 pandemic period as a basis for assessing medical service demands. It analyzes residents’ healthcare-seeking behaviors and the supply–demand ratio of medical resources, establishing a supply–demand ratio indicator for medical resources. (2) Additionally, from the spatial layout perspective, we set up three evaluation indicators, namely, the coverage, accessibility, and equity of medical resources, and make improvements. Specifically, different medical service coverage indicators are set according to the different medical purposes of residents and the functions of hospitals at various levels. In the accessibility indicator, we consider different modes of transportation and the impact of residents’ ages on the accessibility results. The equity indicator takes into account both population and geographical factors. Using an objective weighting method, their respective weights are calculated [24,25,26]. Ultimately, this paper develops a rational evaluation model to assess medical resources’ spatial allocation and analyzes the rationality of the medical facilities in the study area. It can offer recommendations for future city planning [27].

## 2. Materials

### 2.1. Study Area

**Figure 1:**
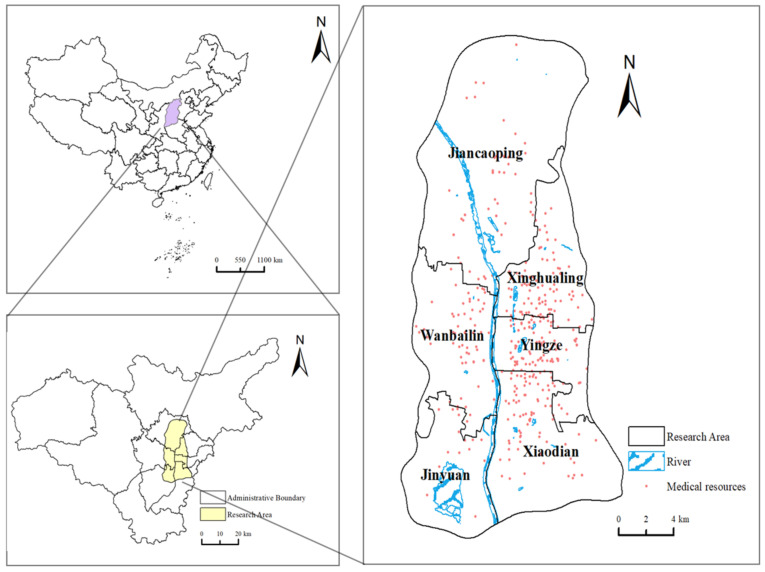
The study area of Taiyuan.

Taiyuan, the capital of Shanxi Province, is currently divided into 6 districts, 3 counties, and 1 county-level city. Taiyuan is the political, economic, cultural, and international center of Shanxi Province and is also one of China’s important energy and heavy industry bases. This article selects the central urban area of Taiyuan City in Shanxi Province as the research area. The scope of Taiyuan’s central urban area is defined in the “Taiyuan City General Urban Planning (2011–2020)”, which includes parts of the six urban districts. It is surrounded by an encircling expressway, covering an area of about 334 square kilometers. The region covers most of Taiyuan City’s population and medical resources, as shown in Figure 1.

### 2.2. Data Sources and Data Pre-Processing

#### 2.2.1. Medical Data

The medical resource data for the central urban area of Taiyuan City were acquired from the official website of the Taiyuan Health Commission’s public announcement in 2022 (https://wjw.taiyuan.gov.cn/, accessed on 1 November 2023) and the official statistics of the Taiyuan City Geographic Information Public Service Platform in 2023 (https://shanxi.tianditu.gov.cn/, accessed on 1 November 2023). A total of 379 medical facilities were collected, including 24 tertiary hospitals. Detailed information, such as hospital names, hospital levels, hospital categories, and the number of planned beds, was also extracted. The latitude and longitude coordinates of each hospital were obtained using the Amap geocoding API.

#### 2.2.2. Population Data

At present, traditional residential agglomerations, such as communities, serve as the primary scale for most studies on the rational allocation of medical resources [28]. However, this approach presents several challenges: (1) Difficulty arises in accurately counting the number of residents within communities due to high population mobility. Consequently, household information often remains outdated, resulting in varying qualities of population data. (2) Some large communities encompass vast areas, leading to residents in different units of the same community being widely dispersed. Using the geometric center of these communities as the reference point for residential locations may result in significant errors when calculating travel time [29,30,31]. In this study, we adopted spatial grids as the research unit and utilized the midpoint of each grid as the residential area, in contrast to the traditional method of using the geometric centers of communities. Additionally, employing spatial grids with dimensions of 1 km × 1 km as the smallest spatial unit allowed for more precise spatial sampling and reduced internal errors on the demand side. Building upon this framework, we extracted the 2020 WorldPop 100 m × 100 m population grid data (https://hub.worldpop.org/, accessed on 15 July 2023). The population raster data were populated into each spatial grid. For raster cells situated between two grids, we used the area-weighting method to allocate the population. This approach ultimately provided more precise population distribution data for each spatial grid.

#### 2.2.3. Road Network Data

The road network data for the study area were sourced from OpenStreetMap (https://www.openstreetmap.org/, accessed on 21 August 2023). Based on the designed speed regulations for roads of different categories outlined in the “Urban Road Engineering Design Standards” (CJJ 37-2012) [32], the road network was classified and edited. The types of road networks and the designed speeds of vehicles in this article are shown in Table 1.

In addition to driving, there are various modes of travel chosen by residents. This article selected walking and cycling as two common modes of transportation, with the cycling speed set at 12.5 km/h and the walking speed at 5 km/h [33].

#### 2.2.4. Dianping Data

Dianping, a well-known third-party consumer rating website, has a large number of users browsing and writing comments on various life facilities and shops. It benefits from a large volume of data and strong objectivity, allowing for the extraction of valid resident demand information (https://www.dianping.com/, accessed on 21 August 2023). For this study, medical resource comment data spanning from 1 January 2020 to 31 December 2022 were extracted, covering the continuous period of the COVID-19 pandemic and serving as demand information for various medical resources during this crisis. To ensure data quality, we cleaned the data by removing duplicate and invalid comment reviews. A total of 594 pieces of medical-resource-related demand information were summarized and visualized by grid units.

#### 2.2.5. Public Transportation Data

Public transportation, as an important component of urban transit, plays an indispensable role in the functioning of cities. The public transportation system in the central urban area of Taiyuan mainly includes buses and subways. Due to the current urban planning, only one subway line is in operation, with a limited overall capacity. Therefore, this study focused on buses as the subject of public transportation research. By utilizing the Amap API (http://lbs.amap.com, accessed on 24 July 2024), bus stop data, bus route data, and the average speed of each bus route in the central urban area of Taiyuan were extracted for February 2023 to provide data support for the accessibility analysis of public transportation. In the study area, a total of 754 bus stops and 429 bus routes were collected, with the overall average speed of buses calculated to be 15.01 km/h. In subsequent experiments, bus stop data and residential point data were spatially overlaid, considering any residential areas within the same grid as having public transportation access. The route data served as the bus network, and the bus speed was set at 15.01 km/h.

## 3. Methods

### 3.1. Technology Frame

**Figure 2:**
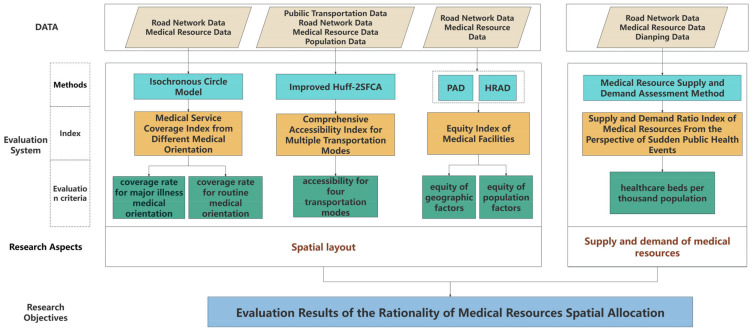
Technology frame of this study.

The technical framework employed in this study is depicted in Figure 2.

First, the central urban area of Taiyuan City served as the research area. An innovative index system was constructed to assess the rationality of medical resources’ spatial allocation. It comprised four indicators categorized into two aspects: the spatial layout and the supply and demand of medical resources. Next, each of the four indicators was individually computed: (1) Medical resources and the road network were utilized to establish an Isochronous Circle Model between residential areas and medical facilities via a network analysis. This involved setting up criteria for major illness medical orientation and routine medical orientation, analyzing residents’ medical orientations and travel behaviors, and calculating the medical service coverage rate index from these different perspectives. (2) The Huff-2SFCA method was improved to calculate a comprehensive accessibility index for multiple modes of transportation. (3) The Population Agglomeration Degree (PAD) and Health Resource Agglomeration Degree (HRAD) were calculated to analyze the equity index of medical facilities from both geographical and population perspectives. (4) Data from Dianping, population data, and medical resources were utilized to quantify the demand for medical services and the service capability of medical institutions based on the number of healthcare beds per thousand people. These data were used to calculate the supply and demand ratio index of medical resources from the perspective of sudden public health events. Then, the entropy weighting method was applied to perform a weighted analysis of the above four indicators, establishing an evaluation model for the rationality of medical resource spatial allocation. Finally, the overall evaluation results of the evaluation model were obtained, and a comprehensive analysis of the results was conducted.

### 3.2. Construction of Index System

While referencing existing indicators of the rationality of medical resource spatial allocation and considering the structure of medical facilities and population density allocation in Taiyuan City, this article systematically categorizes the research perspectives on the spatial allocation of medical resources. It innovatively proposes an evaluation model index system for the rationality of medical resource spatial allocation that considers human-centric demands. In the medical service coverage rate index, we finely categorize medical resource types and propose two distinct medical visitation models based on residents’ preferences: major illness medical orientations, focusing on tertiary hospitals as the endpoint of the search; and routine medical orientations, considering all hospitals as potential endpoints. For the accessibility index, we use the Huff-2SFCA method to calculate the accessibility of medical resources. On this basis, we make specific improvements to the method, including using a Gaussian function as the distance decay function; a comprehensive accessibility value is calculated using four common modes of transportation; and an independent catchment area size and selection weights are tailored for the elderly population. In the equity index, PAD and HRAD are calculated to analyze the equity index of medical facilities from both geographical and population perspectives. In the Supply and Demand Ratio Index, we explore the allocation of medical demands during public health emergencies, using public comment data from Dianping to simulate the population with medical demands. This innovative approach offers a more direct and empirical assessment of actual medical demands during crises.

The first layer is the objective layer, namely, the evaluation model index system for the rationality of medical resource spatial allocation; the second layer is the criterion layer; and the third layer is the indicator layer, which includes various specific indicators corresponding to each criterion layer, as shown in Table 2.

### 3.3. Index Calculation

#### 3.3.1. Medical Service Coverage Rate Index from Different Medical Orientations

Given the diverse functions of hospitals at various levels, tertiary hospitals play an irreplaceable role in dealing with complex and major diseases, while lower-level hospitals, health posts, and community hospitals can fully meet people’s daily medical orientations [34]. Therefore, this study delineates the following criteria based on the purpose of residents’ medical treatments: (1) major illness medical orientations with tertiary hospitals as the search destination and (2) routine medical orientations with all hospitals as the destination. Using an ArcGIS network analysis, an OD cost matrix for medical visits is established, capturing residents’ travel times to the nearest hospital for analysis. Subsequently, an isochrone model is constructed, and a weighted analysis of these scenarios is conducted to derive the medical service coverage rate index. In this paper, based on the studies of Du et al. [35], Nguyen et al. [36], and Wang et al. [37], we find that the overall frequency of residents using basic healthcare is much higher than that of major healthcare. Through summarizing and performing a weighted analysis of these research results, we set the weights for residents’ major illness medical orientations and routine medical orientations to 33.7% and 66.3%, respectively.

(1) $$C_{i}=F_{i}\times W_{F}+M_{i}\times W_{M}$$

where $C_{i}$ represents the medical service coverage rate at habitation locations  i, $F_{i}$ indicates whether the isochrone for routine medical orientation covers locations i, and $M_{i}$ indicates whether the isochrone for major illness medical orientation covers locations  i, with the locations  i being covered marked as 1, or otherwise marked as 0; $W_{F}\;{\mathrm{and}\;W}_{M}$ represent the choice weights for routine medical orientations and major illness medical orientations, respectively.

#### 3.3.2. Comprehensive Accessibility Index for Multiple Transportation Modes

In recent years, the Two-Step Floating Catchment Area (2SFCA) method has gained widespread adoption for its comprehensive approach and strong operational capabilities in analyzing the accessibility of public service facilities [38]. However, the 2SFCA method is not suitable for all scenarios, and an appropriate extension should be chosen based on the characteristics of each experimental area. The city’s six urban districts have undergone rapid urbanization and exhibit developed economic profiles, leading to a geographical clustering of hospitals. Consequently, when examining residents’ medical-seeking behavior, it is imperative to consider the competitive dynamics among hospitals within the analysis framework [39,40]. To address this challenge, this paper uses the Huff extension of the 2SFCA method (Huff-2SFCA). This extension accounts for the influence of the facility supply scale on residents’ travel preferences, quantifies the competitive interactions among supply locations, and effectively models residents’ willingness to travel for medical services. The methodology is outlined as follows.

Step 1: Calculating the selection probability between habitation locations and medical facilities to evaluate the competitive effect among multiple facilities within the same demand nodes search radius:

(2) $$Prob_{ij}^{H}=\frac{S_{j}f(d_{ij})}{\sum_{k\in \{d_{ik}\leq d_{0}\}}S_{k}f(d_{ik})}$$

(3) $$f(d_{ij})=\left\{\begin{array}{l} G(d_{ij})=d_{ij}^{-\beta },d_{ij}\leq d_{0}\\ 0,d_{ij}>d_{0} \end{array}\right.$$

where $d_{\mathrm{ij}}$ represents the time from locations  i to location j , i represents habitation location  i, $d_{0}$ represents the search radius (i.e., catchment size), j represents the facility location j, $S_{j}$ represents the service level of the facility, where this paper uses the number of hospital beds as a proxy, $S_{k}$ represents the service level of some facility k within the search radius, ${f(d}_{\mathrm{ij}})$ represents the distance decay function between i and j, and β represents the impedance coefficient of travel time.

Step 2: Focusing on the medical facility j with $d_{0}\;$ as the search radius, all habitation locations q are searched within the search range, and the supply–demand ratio $R_{j}$ is calculated for each medical facility j:

(4) $$R_{j}=\frac{S_{j}}{\sum_{k\in \{d_{kj}\leq d_{0}\}}Prob_{qj}^{H}P_{q}f(d_{qj})}$$

where ${\mathrm{Prob}}_{\mathrm{qj}}^{H}$ represents the probability of residents choosing to travel from the habitation location q to the medical facility j and $P_{q}$ represents the population number of the location q.

Step 3: Focusing on the habitation location i with $d_{0}$ as the search radius, all medical facilities j are searched within the range, and the supply–demand ratios $R_{j}$ of each demand node i are aggregated to obtain the node’s accessibility $A_{i}^{F}$.

(5) $$A_{i}^{F}=\sum_{j\in \{d_{ij}\leq d_{0}\}}Prob_{ij}^{H}R_{j}f(d_{ij})$$

Based on the Huff-2SFCA model, this paper proposes three improvements:

1. Improvement of the distance decay function.

In the Huff-2SFCA method, the distance decay function used is a power function. Its characteristic is that accessibility decreases faster with an increase in distance at short distances. It is suitable for scenarios such as disaster emergency evacuation [41] and parks [42], where individuals’ preferences sharply decline with distance, and distance itself plays a dominant role. Conversely, the Gaussian function exhibits an “S”-shaped decay pattern. Compared to the power function, it features a smoother overall decay rate. In the complex urban environment, factors such as hospital grade and expert authority are of greater significance in residents’ selection of medical facilities, thereby diminishing the influence of distance. Consequently, the Gaussian function proves to be more applicable to actual medical scenarios. Hence, this paper chooses the Gaussian function to replace the power function. The formula is as follows:

(6) $$f(d_{ij})=\left\{\begin{array}{l} G(d_{ij})=\frac{e^{-\frac{1}{2}\times (\frac{d_{ij}}{d_{0}})}{\;}^{2}-e^{-\frac{1}{2}}}{1-e^{-\frac{1}{2}}},d_{ij}\leq d_{0}\\ 0,d_{ij}>d_{0} \end{array}\right.$$

2. Improvement of transportation modes.

The choice of transportation mode significantly impacts residents’ mobility, with notable variations observed between different modes within the same timeframe. Driving is widely acknowledged as the swiftest option for long-distance travel, with over 60% of residents opting for it for longer journeys. Conversely, walking is favored for shorter distances due to its convenience, while cycling emerges as a viable alternative, particularly for households without cars or in areas with limited commuting options. In addition, public transportation is the primary mode of travel for many urban residents, especially for low-income groups, students, and elderly people. Consequently, this paper considers four primary modes of transport: driving, cycling, walking, and taking the bus. Referring to the research by Du et al. [43] on urban travel modes, this study assigns the following weights to each mode of transport: 20% for driving, 30% for walking, 10% for cycling, and 40% for public transportation. Given that the accessibility outcomes derived from different transportation modes exhibit varying ranges, a normalization process is initially applied to the accessibility values. Subsequently, the accessibility values for the four transport modes are weighted to yield a comprehensive spatial accessibility measure. The formula is presented below:

(7) $$A_{i}^{F}=A_{i-d}^{F}\times B_{d}+A_{i-w}^{F}\times B_{w}+A_{i-b}^{F}\times B_{b}+A_{i-p}^{F}\times B_{p}$$

where $A_{i-d}^{F}$, $\;A_{i-w}^{F}$, $\;A_{i-b}^{F}$, and $\;A_{i-p}^{F}$ represent the accessibility by driving, walking, and cycling, respectively. $B_{d},$ $B_{w}$, $B_{b}$, and $B_{p}$ represent the choice weights for driving, walking, cycling, and public transportation, respectively.

3. Improvement of catchment size.

Given the notable variations in travel patterns for medical visits among different age demographics, particularly considering the mobility challenges faced by elderly people due to physical limitations, it becomes imperative to adjust the catchment size for medical accessibility across age groups. According to the “Taiyuan City Elderly Rights Protection Measures”, approximately 18.4% of Taiyuan’s population is elderly. Consequently, this study conducts separate analyses for the elderly and non-elderly populations concerning walking as a mode of travel, with distinct catchment size parameters. For the elderly population, the walking catchment size is set to 80% of the standard value to account for reduced mobility. According to Du’s research [43], buses, cars, and walking are the main modes undertaken by elderly people to seek medical treatment. However, for bus and car travel, the factor affecting travel time is the speed of the vehicle, not the mobility conditions of elderly people. Therefore, we only differentiate the catchment area for walking between the elderly and non-elderly populations, without additional processing for the other modes of travel. The refined accessibility formula is presented below:

(8) $$Prob-o_{ij}^{H}=\frac{S_{j}f(d_{ij},d_{1})}{\sum_{k\in \{d_{ik}\leq d_{0}\}}S_{k}f(d_{ik},d_{1})}$$

(9) $$R_{j}=\frac{S_{j}}{\sum_{k\in D_{0}}\left(Prob_{qj}^{H}P_{q}f(d_{qj},d_{0})+Prob-o_{qj}^{H}P_{q}f(d_{qj},d_{1})\right)}$$

(10) $$A_{i-w}^{F}=\sum_{j\in D_{0}}(Prob_{ij}^{H}R_{j}f(d_{ij},d_{0})+Prob-o_{ij}^{H}R_{j}f(d_{ij},d_{1}))$$

where $d_{0},\;\;d_{1}$ represent the accessibility by driving, walking, and cycling, respectively.

Finally, the comprehensive accessibility value for multiple modes of transport is:

(11) $$A_{i}^{F}=A_{i-d}\times B_{d}+A_{i-w}\times B_{w}+A_{i-b}\times B_{b}+A_{i-p}\times B_{p}$$

where, $d_{0},\;d_{1}$ represent the size of the catchment for the non-elderly and elderly populations, respectively. $Prob-o_{\mathrm{ij}}^{H}$ represent the probability of choosing to go from the habitation location k to the medical facility j for the elderly population.

#### 3.3.3. Equity Index of Medical Facilities

By calculating the Population Agglomeration Degree (PAD) and Health Resource Agglomeration Degree (HRAD) indices, we can effectively incorporate population and geographic factors to assess spatial equity indicators. The PAD reflects the degree of population concentration in an area that occupies 1% of the total area relative to the entire region’s population. The formula for its calculation is as follows [44,45].

(12) $$PAD_{i}=(\frac{P_{i}}{P_{n}})\times 100\%÷(\frac{a_{i}}{a_{n}})\times 100\%$$

where ${PAD}_{i}$ represents the Population Agglomeration Degree of the grid i, $P_{i}$ is the population within the grid i, $P_{n}$ is the total population in the study area, $a_{i}$ is the land area of a grid i, and $a_{n}$ is the total land area of the study area.

Similarly, applying the concept of the Population Agglomeration Degree to assess health resources leads to the calculation of the Health Resource Agglomeration Degree (HRAD), which reflects the concentration of medical resources in a region occupying 1% of the area relative to the entire region’s medical resources. This is commonly used in evaluating the equity of health resource allocation. The calculation formula is as follows:

(13) $$HRAD_{i}=(\frac{HR_{i}}{HR_{n}})\times 100\%÷(\frac{a_{i}}{a_{n}})\times 100\%$$

where ${HRAD}_{i}$ represents the Health Resource Agglomeration Degree of the grid i  and $\;{HR}_{i}$ is the number of medical institutions in the grid.

From a geographic perspective, if ${HRAD}_{i}$ is greater than 1, it indicates that the medical resources within the designated 1% area exceed 1% of the total health resources, suggesting a higher density of resources and greater equity in geographic allocation within that area. Conversely, if ${HRAD}_{i}$ is less than 1, it indicates a deficiency of health resources in that area, suggesting lower geographic equity and a need to augment health resources.

From a population perspective, if ${HRAD}_{i}$ minus ${PAD}_{i}$ is equal to 0, it suggests that the ratio of health resources to the population within the 1% area is balanced, indicating equitable allocation based on population factors. A positive difference suggests that the per capita health resources exceed the regional average, indicating a better equity in allocation based on population factors. Conversely, a negative difference indicates a deficiency of health resources, reflecting a poorer equity. The formula representing this relationship is as follows:

(14) $$FG_{i}=HRAD_{i}$$

(15) $$FP_{i}=HRAD_{i}-PAD_{i}$$

where, ${FG}_{i}$ represents the equity of the geographic factors index within the grid i and ${FP}_{i}$ represents the equity of population factors index within the grid i.

#### 3.3.4. Supply and Demand Ratio Index of Medical Resources from the Perspective of Sudden Public Health Events

In accordance with the “Guidance Principles for the Planning of Medical Institutions (2021–2025)” [46], the number of healthcare beds per thousand people stands as a pivotal indicator for the establishment of medical facilities outlined in the Principles. It effectively reflects the medical service demand and capability of medical institutions. This study uses the number of healthcare beds per thousand people to quantify the supply–demand ratio index. Additionally, considering the recent outbreak of COVID-19 and other emergencies that pose significant risks to public health, characterized by their unpredictability and potential for widespread health impacts, it is crucial to learn from past experiences, such as those during the COVID-19 pandemic. Investigating the healthcare-seeking patterns of residents during such periods and preparing resource allocations and emergency plans in advance based on the existing medical resources during safe periods are essential. Therefore, studying the supply and demand relationship of medical resources from the perspective of emergency medical events is very necessary [47,48]. To address this, we use each spatial grid area as the basic research unit and integrate medical resources comment data from Dianping during the COVID-19 pandemic period as a proxy for medical demand data. Specifically, we collect the number of comments for each medical institution on Dianping and aggregate the total numbers of comments within each spatial grid, recording them as the medical needs arising during the COVID-19 pandemic period for that grid. We then proportionally allocate the population data within each grid area based on the demand ratio for medical services, simulating the potential number of people seeking medical treatment during special medical events (such as the COVID-19 pandemic). The purpose of this approach is to assess whether the capacity of each medical institution is sufficient to handle the peak demand during extreme events. The formulas are shown as follows (16) and (17).

(16) $$PN_{i}=\frac{D_{i}}{D_{t}}\times P$$

where $D_{t}$ represents the total number of public comments on medical resources across all grids, $D_{i}$ represents the number of public comments on medical resources within grid i, i represents the grid number, P represents the total population within the study area, and ${PN}_{i}$ represents the population in the grid i  allocated according to medical demand.

(17) $$V_{i}=\frac{1000}{PN_{i}}\times B_{i}$$

where $V_{i}$ represents the number of healthcare beds per thousand people in the grid i. $B_{i}$ represents the total number of medical beds in the grid i.

### 3.4. Index Weighting Methodology

This paper establishes an evaluation model aimed at assessing the rationality of the spatial allocation of medical resources through four distinct indicators. However, the inherent importance of each indicator varies and should be calculated based on relevant mathematical theories. The entropy weight method, an objective approach commonly used in comprehensive evaluation schemes, is employed to determine the weights of these indicators. Unlike subjective methods, such as manual weighting, the entropy method reduces the potential for human error and provides a higher accuracy and objectivity, thus enhancing the interpretation of the results [49]. The calculation method is as follows:

1. Normalization of the Indicators: The purpose of normalization is to remove the dimensions:

(18) $$Y_{ij}=\frac{X_{ij}-min(X_{i})}{max(X_{i})-min(X_{i})}$$

2. Calculation of the Proportion of Each Indicator for All Samples:

(19) $$p_{ij}=\frac{Y_{ij}}{\sum_{i=1}^{n}Y_{ij}},i=1,\ldots ,n,j=1,\ldots ,m$$

3. Calculation of the Information Entropy for Each Indicator:

(20) $$E_{j}=-\ln{\left(n\right)}^{-1}\sum_{i=1}^{n}p_{ij}\ln p_{ij}$$

4. Calculation of the Weights for Each Indicator:

(21) $$W_{j}=\frac{1-E_{j}}{m-\sum E_{j}}$$

where m is the total number of indicators, n is the number of samples, $X_{\mathrm{ij}}$ is the value of the indicator i of the sample j, $Y_{\mathrm{ij}}$ is the normalized result, $p_{\mathrm{ij}}$ is the proportion of the indicator i in the sample j, $E_{j}$ is the entropy of the indicator j, and $W_{j}$ is the weight of the indicator j.

Finally, the calculated weights are used to compute the comprehensive evaluation index of the medical resource spatial allocation rationality evaluation model, as shown in Formula (22):

(22) $$I_{i}=C_{i}\times W_{C}+A_{i}^{F}\times W_{A}+FG_{i}\times W_{G}+FP_{i}\times W_{p}+V_{i}\times W_{V}$$

where $I_{i}$ represents the medical resource allocation rationality evaluation index of the grid i, $C_{i}$, $A_{i}^{F}$, ${FG}_{i}$, ${FP}_{i}$, and $V_{i}$ represent the indices of each indicator for the grid  i, and $W_{C}$, $W_{A}$, $W_{G}$, $W_{P}$, and $W_{V}$ represent the weights of the indicators in the medical resource allocation rationality evaluation model, respectively.

## 4. Results

### 4.1. Analysis of Index Terms of the Evaluation Model of Rationality of Medical Resources Spatial Allocation

#### 4.1.1. Analysis of Medical Service Coverage Rate Index from Different Medical Orientations

Referencing the “15-Minute Convenience Zone” proposed in the “Shanxi Province’s Pilot Implementation Plan for Advancing the Construction of Urban 15-Minute Convenience Life Circles (2022–2024)” [50], this article adopts a 15-min walking time as the travel time threshold for residents’ routine medical orientation. However, for tertiary hospitals, it is difficult to determine a clear standard for search radii based on the existing research. This necessitates field research tailored to the characteristics of medical facilities in the study area [51]. Therefore, this paper uses the ArcGIS Network Analyst function to calculate the shortest walking times from each residential point to all hospitals and tertiary hospitals, using 15-, 30-, 45-, and 60-min thresholds, to determine the optimal threshold choice and visualize residents’ travel times to medical facilities through isochrone maps.

The statistical results, presented in Table 3, indicate that, at a 15-min travel time, walking isochrones cover only 6.61% of residential areas and 4.33% of the total area. Extending the threshold to 30 min, isochrones for major illness medical orientations cover about 25% of residential points and areas. Further expanding to 60 min, 56% of the urban area and 55.92% of the residential points are included in the isochrones. At this point, the expansion of the isochrones slows down with time. Considering factors such as residents’ physical endurance, 60 min is set as the time threshold for residents’ major illness medical orientations.

**Figure 3:**
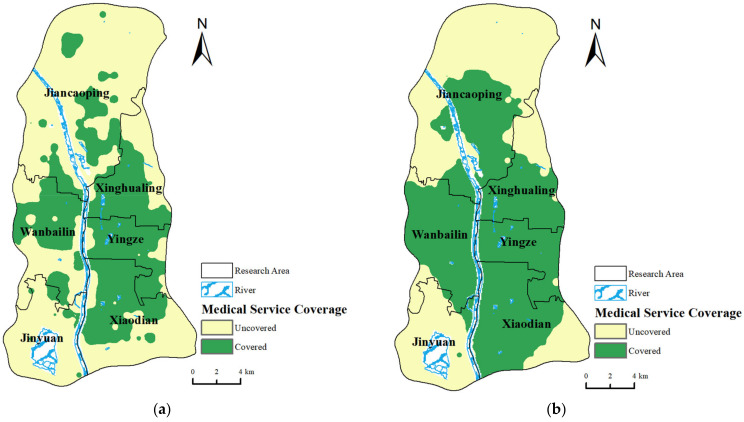
Results of isochrone coverage rate for two types of medical orientations. (**a**) Coverage rate results for routine medical orientations and (**b**) coverage rate results for major illness medical orientations.

**Figure 4:**
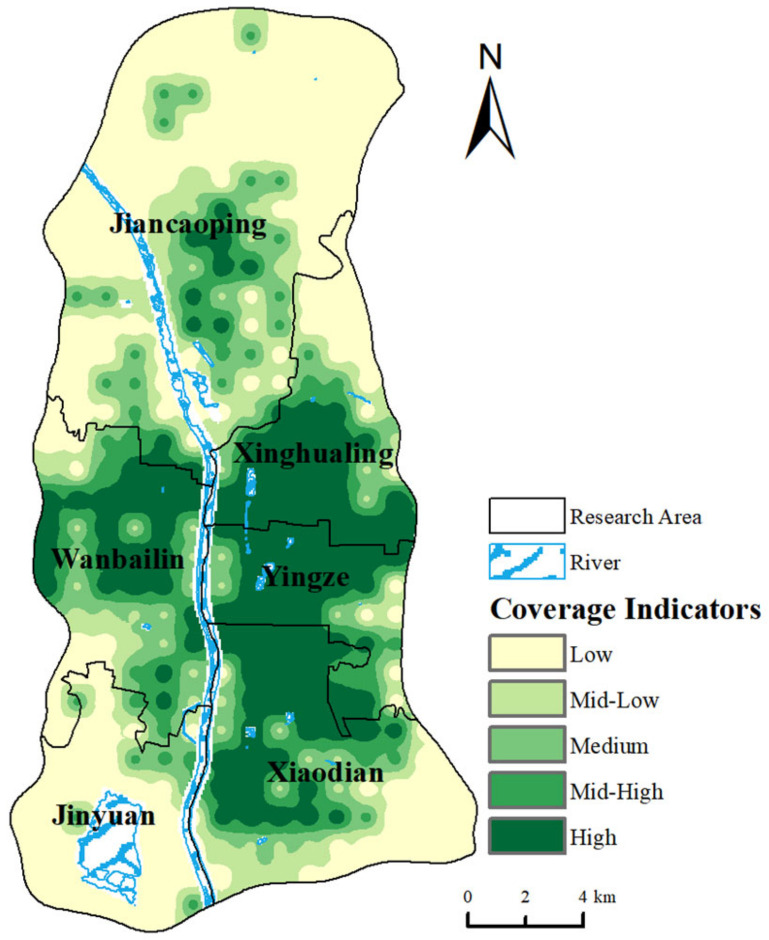
Results of medical service coverage rate indicators from different medical orientations.

Combining the area coverage rates and population coverage rates for the same time in two types of medical orientations, the area of the isochrones for tertiary hospitals, due to their larger search threshold, is bigger than that for all hospitals. In terms of routine medical orientations, the areas covered by the 15-min isochrones mainly concentrate around the intersection of Xinghualing District, Yingze District, Xiaodian District, and Wanbailin District (Figure 3a). For major illness medical orientations, the residential points within the 60-min isochrones are mainly distributed in roughly the same areas as those for routine medical orientations, with the only difference being that the center of Jiancaoping District is also included in the service range of tertiary hospitals due to some tertiary hospitals concentrated in the central area of Jiancaoping District, resulting in shorter travel times to medical services nearby (Figure 3b). Based on the two types of resident medical orientations, this paper extracts the proportion of residential points covered by the isochrones, assigns a value of 1 to residential points within the isochrones and 0 to areas outside, and calculates the medical service coverage rate index based on a 50% weight for each type of medical orientation. The results are classified using the natural breaks method, as shown in Figure 4.

From Figure 4, it can be seen that the overall trend of the medical service coverage rate index values aligns closely with the isochrone coverage results for residents’ major and routine medical orientations. Areas with higher coverage rates are concentrated in the central region of the study area, the south–central area of Jiancaoping District, and the northern area of Xiaodian District. This result reflects that the nearby tertiary and general hospitals are numerous and capable of providing comprehensive medical services.

#### 4.1.2. Analysis of Comprehensive Accessibility Index for Multiple Transportation Modes

**Figure 5:**
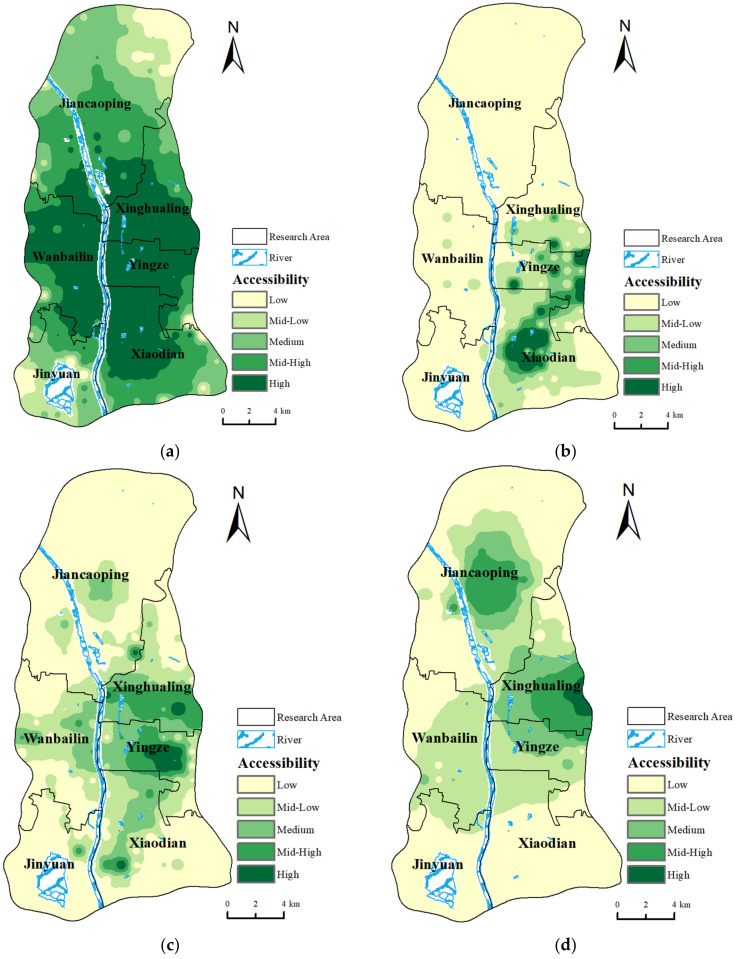
Results of accessibility for four transportation modes. (**a**) Driving accessibility; (**b**) riding accessibility; (**c**) walking accessibility; and (**d**) public transportation accessibility.

This article adopts a 30-min accessibility search threshold and utilizes the ArcGIS Network Analyst function, applying the improved Huff-2SFCA model to assess the accessibility of various transportation modes in Taiyuan City. The results are illustrated in Figure 5.

This paper uses the natural breaks classification method to categorize the accessibility results of various modes of travel into five levels: low, mid–low, medium, mid–high, and high. The natural breaks classification method is based on the natural distribution characteristics of the data, identifying significant changes in the data values to determine the classification breaks. Compared to the same standard classification method, this approach has the advantage of independently classifying the accessibility values of each mode of transportation, better reflecting the accessibility distribution characteristics of each mode. It avoids obscuring the actual distribution characteristics and advantages of certain modes of travel due to differences in accessibility ranges, making the classification results more realistic and interpretable. Accessibility in the context of medical resource allocation is defined as the ease with which residents can obtain medical services. The accessibility trends across the four transportation modes exhibit a similar pattern, characterized by a gradual decrease from the city center outward. The highest accessibility is mainly concentrated along the banks of the Fen River in the central area, notably in Yingze District, the southern part of Xinghualing District, the northern part of Xiaodian District, and select areas in the eastern segment of Wanbailin District. This region serves as the urban epicenter of Taiyuan, boasting ample medical facilities and excellent transportation infrastructure, thus ensuring a superior accessibility.

However, the accessibility levels vary slightly depending on the mode of travel:

Driving: The highest accessibility values are observed in Yingze District and its adjacent areas, forming a prominent high-accessibility zone spanning the Fen River and situated in the central–southern sector of the study area, covering approximately one-third of the total area. The outer layers of accessibility exhibit a gradual decline with an increasing distance, with the northernmost region experiencing a relatively lower accessibility due to its distance from the urban core, resulting in fewer medical resources and less convenient transportation (Figure 5a).

Cycling: A high accessibility is delineated into two main regions. The first region extends outward in a scattered dot pattern, closely spaced and interconnected to form a high-accessibility area, located between Yingze District and the southern part of Xinghualing District, centered around Taiyuan Bus Station. High accessibility points are near the middle section of Caiyuan Street, TaiTie Garden, and south of Longtan Park. The second region is in the central–western area of Xiaodian District, with a large range of high accessibility values centered around the intersection of Tiyu Road and Keji Street (Figure 5b).

Walking: High-accessibility areas are primarily concentrated in Yingze District and Xinghualing District. Additionally, there are several areas with high-accessibility points, such as the southwestern area of Xiaodian District, the eastern area of Jinyuan District, and the central area of Jiancaoping District near Taigang. These areas are close to large commercial centers, boasting convenient transportation options and numerous hospitals, thus ensuring a high accessibility (Figure 5c).

Public transportation: High-accessibility areas are concentrated in the central part of Jiancaoping District and the south–central part of Xinghualing District, forming two highly accessible clusters centered around these areas. Regions with relatively high accessibility values cover most of Yingze District and Wanbailin District, while the public transportation accessibility in the two southern districts of Taiyuan remains slightly lower (Figure 5d).

**Figure 6:**
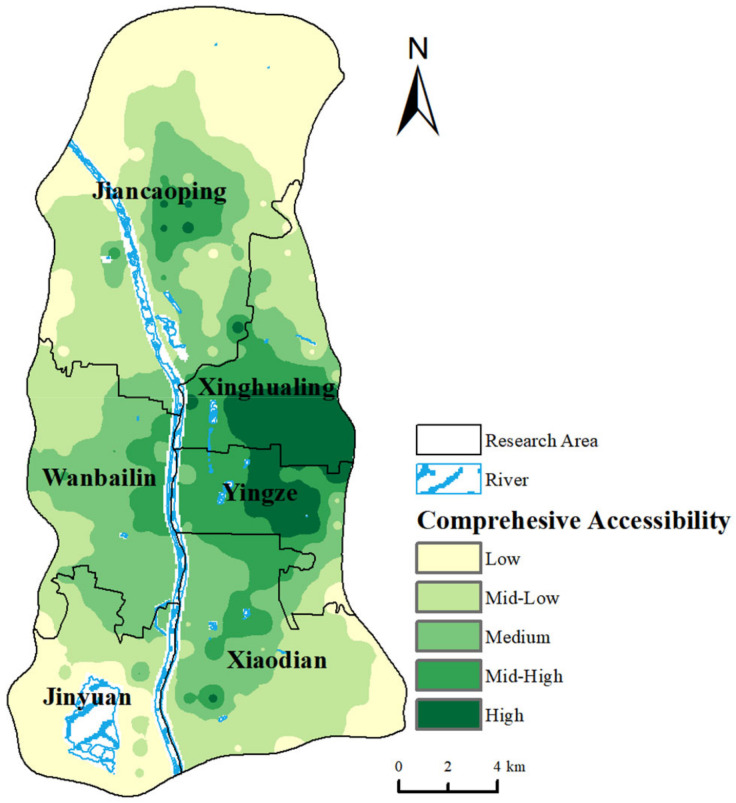
Results of comprehensive accessibility indicators for multiple transportation modes.

The accessibility values of the four transportation modes are normalized and linearly weighted based on the predetermined mode weights, obtaining a composite accessibility value for medical services, which is depicted in Figure 6. The results indicate that the allocation trend of the comprehensive accessibility values is the highest in the central and western urban core areas, decreasing in a ring-shaped pattern outward. The high accessibility value centers are centered at the junction of Yingze District and Xinghualing District. Conversely, Jiancaoping District and Jinyuan District, situated along the north–south axis, exhibit relatively lower overall accessibility levels.

#### 4.1.3. Analysis of Equity Index of Medical Facilities

**Figure 7:**
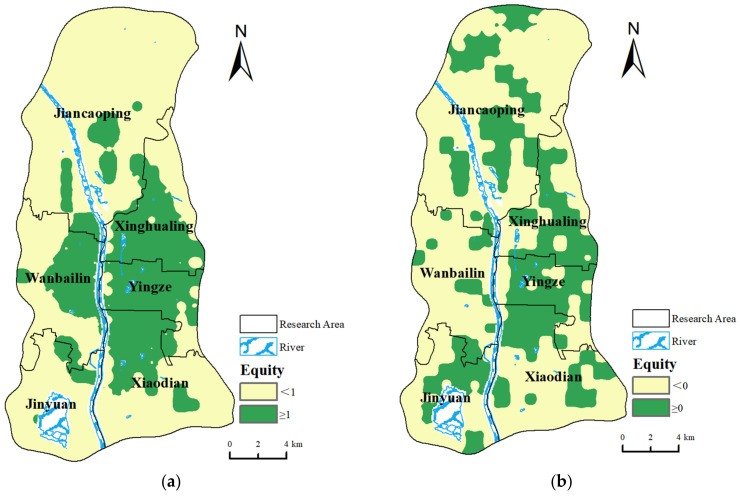
Results of equity indicators for medical facilities. (**a**) Equity in geographic factors and (**b**) equity in population factors.

This article provides a quantitative assessment of the equity of medical facilities within each spatial grid using two key indicators: PAD and HRAD, examining the equity of medical resources from population and geographic perspectives. Data on medical institutions, population figures, and geographical areas of each district were compiled to calculate the equity index, with the results illustrated in Figure 7.

Analysis of geographic perspectives: ${FG}_{i}$ assesses the allocation of medical resources based on geographic factors. Areas where the ${FG}_{i}$ is greater than 1, indicating an equitable allocation of medical institutions, are primarily found in Xinghualing District, Yingze District, the northern part of Xiaodian District, and the eastern part of Wanbailin District. This demonstrates that the quantity of medical facilities in these areas is sufficient relative to their geographic size. Notably, more than 80% of the areas of the Xinghualing and Yingze districts have an ${FG}_{i}$ greater than 1, indicating the highest overall equity. While some areas within the Wanbailin and Xiaodian districts show a good equity, the overall allocation of medical facilities in these districts is not entirely balanced. Conversely, the Xinghualing and Jinyuan districts have numerous areas with ${FG}_{i}$ indices less than 1, indicating an imbalance in the ratio of medical facility numbers to the geographic area and suggesting a need for additional medical facilities (Figure 7a).

Analysis of population perspectives: ${FP\;i}_{i}$ reflects the allocation of medical resources based on population factors. Areas with an ${FP\;i}_{i}$ greater than 0 are considered to have a fair allocation, while those with less than 0 are unfair. The overall trend is similar to that observed with the geographic factors. The Xinghualing and Yingze districts have a high proportion of areas with an ${FP\;i}_{i}$ greater than 0, indicating a well-stocked reserve of medical resources. The Jiancaoping and Jinyuan districts show a good population fairness, where medical institutions are distributed reasonably in terms of population numbers. Other areas generally show a mismatch between medical resource allocation and population numbers, indicating a poor fairness (Figure 7b).

Overall Conclusion: From both the population and geographic perspectives, the Yingze and Xinghualing districts exhibit a good performance for both indicators, owing to their developed economies, convenient transportation, and the fact that their areas and population sizes are relatively small compared to other districts while having numerous medical resources This leads to a fairer allocation of medical resources. However, the remaining four districts present mixed results, with some areas performing well and others poorly, indicating overall deficiencies in the fair allocation of medical resources across the region.

#### 4.1.4. Analysis of Supply and Demand Ratio Index of Medical Resources from the Perspective of Sudden Public Health Events

**Figure 8:**
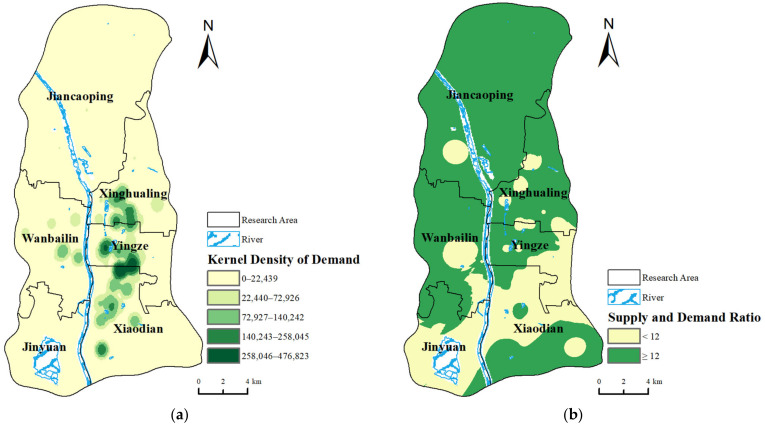
Results of supply and demand ratio index of medical resources. (**a**) Kernel density of demand information and (**b**) results of supply and demand ratio.

Incorporating the medical demand data from the COVID-19 pandemic into the ArcGIS 10.4 software for kernel density estimation and mapping allows for a clear visualization of the medical demands during the emergency across different regions, as shown in Figure 8. Figure 8a displays the kernel density of demand information, revealing that Xiaodian District, Yingze District, and Xinghualing District have the highest concentrations of medical demand, accounting for 38.3%, 29.7%, and 22.1% of the total demands respectively, representing a significant portion of these resident medical demands. Using the number of healthcare beds per thousand people in Taiyuan’s central urban area, which stands at 12 beds per thousand people, as a benchmark, the healthcare beds per thousand people for each grid were analyzed. The results of the medical resources’ supply and demand ratio are presented in Figure 8b.

The supply and demand ratio index quantitatively describes whether the existing medical service capacity can meet residents’ medical demands during sudden public health events. As indicated in the figure, the number of beds per thousand people in most areas of Xinghualing District, Jiancaoping District, Wanbailin District, and Yingze District exceed the overall average, suggesting that these areas have strong medical service capacities and are in a balanced state regarding medical resources’ supply and demand. Conversely, most areas in Xiaodian District and Jinyuan District have supply and demand indices lower than the average, indicating relatively weaker medical service capacities. Overall, the areas with high medical demands, particularly near the core functional zones and in the northern part of the study area, generally have higher supply and demand ratio indices, demonstrating that the existing medical resources in these regions can meet the high medical demands during special periods. However, the capacity for emergency medical services in some southern parts of the study area still requires strengthening to better meet potential demands.

### 4.2. Evaluation and Analysis of Rationality of Medical Resource Spatial Allocation

This paper employs the entropy weight method to normalize the four indicators, eliminate dimensions, compute information entropy, and ultimately determine the weights for each indicator, as outlined in Table 4.

**Figure 9:**
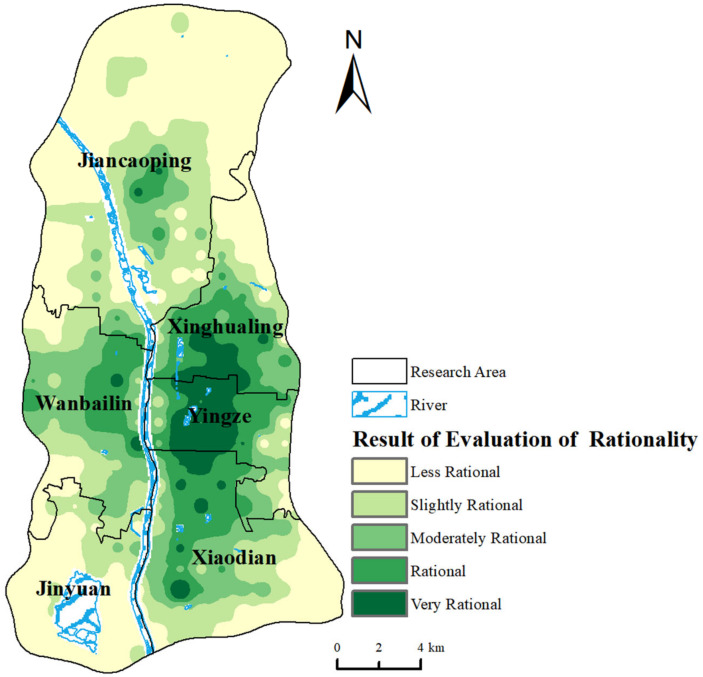
Rationality assessment results of medical resources spatial allocation.

Based on these weights, the five indicator items across the four main indicators are linearly weighted according to Formula (22), resulting in a comprehensive medical resource allocation rationality index. The index is then classified into five levels using the natural breaks classification method, ranging from very rational to irrational, as presented in Table 5. This comprehensive rationality evaluation index is imported into ArcGIS 10.4 and visualized using the Inverse Distance Weighted (IDW) interpolation method, yielding the following results:

As depicted in Figure 9, the medical resource spatial allocation rationality rating exhibits a ‘ring-layer’ allocation pattern. In the core urban area, at the intersection of the Yingze and Xinghualing districts, the rationality of medical resource allocation is notably high. This rationality decreases as one moves outward from the center. This ‘very rational’ area, located in Taiyuan’s densely populated core functional zone, comprises 5.68% of the study area and includes multiple business districts, high levels of economic development, well-established transportation networks, and comprehensive medical facilities. Additionally, the eastern part of Wanbailin District, the northern part of Xiaodian District, and the central part of Jiancaoping District are also categorized as ‘very rational’ zones, situated near major tertiary hospitals and community hospitals that enhance medical service availability. The area rated as rational comprises 14.24% of the total area, spatially surrounding the very rational areas, mostly concentrated in the Xinghualing, Wanbailin, Yingze, and Xiaodian districts. Areas rated as moderately rational, covering 18.95% of the total area, are mainly located in the central and southern parts of Jiancaoping District, central Xiaodian District, parts of Xinghualing District, and Wanbailin District. The two lower ratings, comprising 61.03% of the study area, include 38.73% in areas rated as irrational, where medical resource supply is relatively low, geographically distant from the urban core, mostly in Jiancaoping, Jinyuan Districts, the southern part of Xiaodian District, and the northern part of Xinghualing District.

In summary, the findings reveal significant spatial heterogeneity in the rationality of medical resource allocation within the central urban areas of Taiyuan, showing distinct regional differences among districts and counties (see Figure 9). The rationality of medical resource allocation is influenced by factors such as geographical location, economic development, transportation, and population density. The most rational allocations are found in economically developed and transit-accessible areas such as the Yingze and Xinghualing districts. In contrast, Jinyuan District and Jiancaoping District, located in the north and south, respectively, have fewer medical resources. Due to smaller populations, larger geographical areas, and greater distances from the central district, some areas experience inadequate medical allocation. Additionally, the internal regions of Wanbailin District and Xiaodian District display varying levels of rationality, indicating a relatively uneven allocation of internal rationality (see Figure 3, Figure 4, Figure 5, Figure 6, Figure 7, Figure 8 and Figure 9).

## 5. Discussion

In this study, we evaluated the spatial allocation rationality of medical resources in Taiyuan City using four key indicators across two dimensions: the spatial layout and the supply and demand of medical resources. The indicators are the medical service coverage rate index, accessibility index, equity index, and supply and demand ratio index.

From a spatial layout perspective on the spatial allocation of medical resources, the spatial coverage rate of medical services still requires improvement. Residents’ demands for major medical care at tertiary hospitals and basic healthcare services at all types of hospitals are not fully met. To realize plans like the “15-min convenience living circle”, more attention must be given to the northern and southern parts of the city, where access to medical resources is challenging. This was similar to the findings of Haylee Lane and Li [52,53], who found that medical resources are primarily concentrated in the core areas of the city. Secondly, this study analyzed the accessibility of medical resources regarding four common modes of transportation: driving, cycling, walking, and buses. The results indicated that all residents can access medical services within 30 min when traveling by car. As for public transportation, the high-accessibility areas are centered around Xinghualing District and extend into parts of the Yingze and Xiaodian districts. Notably, for walking and cycling, the highest value of walking accessibility is located in Yingze District, while the highest value of cycling accessibility is in Xiaodian District. This is contrary to the findings of Mao [54], who demonstrated that the overall trend of accessibility for different modes of travel is generally consistent. The primary reason for this difference is that, as travel capability increases, the main factor affecting accessibility gradually shifts from the service capacity of medical resources to the concentration of medical institutions. In Xiaodian District, medical institutions are more concentrated and densely located. Additionally, the introduction of relevant policies can also impact actual accessibility. For example, the Taiyuan city government launched an urban quality improvement project for Yingze Street, which includes widening some pedestrian crossings in Yingze District to promote walking convenience. These results are closely related to the locations of medical facilities and transportation convenience, as well as the scale and attractiveness of hospitals. Then, slight differences in medical service equity are observed from both the population and geographical perspectives. Generally, core urban areas exhibit a higher equity, whereas peripheral areas show lower levels of equity. In the northern Jiancaoping District and southern Jinyuan District, due to their smaller populations, the overall demand for medical resources tends to be lower. As a result, fewer medical resources are sufficient to meet the healthcare demands of most residents in these regions. This conclusion concords with Wu and Wu [55,56]

Regarding the supply and demand of medical resources, this study simulated the supply–demand situation of medical resources during the COVID-19 pandemic. It was found that the residents’ healthcare demands were highest in Xiaodian District, Yingze District, and Xinghualing District, which aligns with the overall findings presented in this study. By calculating the carrying capacity of medical institutions and the number of residents in need, the study verified that most areas could meet these healthcare demands during public health emergencies. Notably, this study expands on the existing literature by incorporating the supply–demand perspective of medical resources. The research indicates that conducting a rationality analysis from the supply–demand perspective of medical resources is meaningful, as it can utilize past experiences to guide relevant departments in making preemptive adjustments and stability assessments based on whether the supply and demand of medical resources are balanced in different regions.

Based on these findings, we can give some suggestions for the improvement of Taiyuan’s spatial allocation of medical resources. Regions with rational resource allocation generally follow a pattern, with a tendency towards central resource concentration. These findings support the objectives outlined in the ‘14th Five-Year Plan for the Construction of a High-Quality and Efficient Medical and Health Service System’, which advocates for the expansion of regional medical center construction areas and the promotion of a balanced development of medical resources. Measures should be implemented to mitigate the excessive concentration of medical resources in the urban core areas. For regions with inadequate medical resources, it is advisable to adhere to the strategy of “selecting hospitals by key diseases and areas by needs, and promoting provincial and departmental cooperation”. Encouraging the redirection of medical resources towards urban peripheries and less populated areas can enhance the medical service levels there and reduce the disparity in medical resources between the city center and peripheral regions.

There are some limitations of this study. In light of the continuous advancements in internet technology and the proliferation of various sensors, the ubiquitous sensing perspective facilitates the tracking of map resources and residents’ travel behaviors, thereby offering enhanced methods for evaluating the rationality of medical facilities. Building upon the aforementioned analysis, this paper proposes several strategies for future improvement: (1) When calculating accessibility, the existing improved Huff-2FSCA algorithm does not take into account the actual ground traffic conditions, such as rush hour congestion and road maintenance. By using the Open Map Path Planning API, it is possible to better simulate the actual travel times for various modes of transportation. (2) Vast data resources from social media can be utilized, such as mobile phone signal data, taxi and bicycle trajectory data, and heat maps. These data sources can improve the spatial estimation accuracy of small-scale population data, thus providing more precise data support for the rationality evaluation model. (3) The economic, social, and natural dimensions of medical data can be incorporated to establish a multi-dimensional index within the rationality evaluation model, making the evaluation results more comprehensive.

## 6. Conclusions

The findings emphasize that the current spatial allocation of medical resources in Taiyuan still has room for improvement. Authorities and policymakers should focus on enhancing the healthcare service capacity in non-core urban areas. This enhancement should not only involve the strategic addition of new healthcare facilities, but also the balanced allocation of different levels of medical institutions within a given region. Overall, this research provides actionable insights and recommendations that can assist in formulating effective policies and strategies aimed at achieving a more rational and equitable spatial allocation of healthcare resources.

Due to the numerous factors influencing the allocation of medical resources in Taiyuan, including economic development level, population density, geographical location, medical policies, and the social security system, these factors are interrelated, mutually restrictive, and affect each other. Therefore, future research will systematically explore the interactions and comprehensive effects of these factors to accurately identify and assess the key determinants of medical resource allocation. This will help in formulating more effective policies and strategies to optimize the allocation of medical resources.

## Figures and Tables

**Table 1:** Vehicle speeds on various types of roads.

Road Class	Expressway	Trunk Road	Secondary Road	Spur Road
Speed of design (km/h)	80	50	40	20

**Table 2:** Evaluation model index system for the rationality of medical resource spatial allocation.

Objective Layer	Criterion Layer	Overall Indicator Layer	Description
Evaluation Model Index System for the Rationality of Medical Resources Spatial Allocation	Spatial Layout	1. Medical Service Coverage Rate Index from Different Medical Orientations $(C_{i}$)	Calculate the service coverage rates of various levels of medical institutions based on the behavior patterns of residents choosing corresponding medical institutions under different disease conditions
		2. Comprehensive Accessibility Index for Multiple Transportation Modes $(A_{i}^{F}$)	Quantify the convenience of residents obtaining medical services through different modes of transportation, including driving, walking, bus, and cycling
		3. Equity Index of Medical Facilities $({\mathrm{FP}}_{i},\;{\mathrm{FG}}_{i}$)	Describe whether the distribution of medical resources and services is equitable among different population groups and geographic regions
	Supply and Demand of Medical Resources	4. Supply and Demand Ratio Index of Medical Resources From the Perspective of Sudden Public Health Events $(V_{i}$)	Assess whether medical resources can meet medical demands during sudden medical events

**Table 3:** Isochrone coverage rate of residents’ travel patterns.

Time (min)			0–15	15–30	30–45	45–60	>60
Walking	Major illness medical orientations $(M_{i}$)	Percentage of residential points	6.61%	20.11%	15.43%	13.77%	44.08%
		Percentage of area	4.33%	21.32%	16.15%	14.20%	44.00%
	Routine medical orientations $(F_{i}$)	Percentage of residential points	39.67%	29.75%	16.53%	7.16%	6.89%
		Percentage of area	37.64%	36.08%	15.21%	6.78%	4.29%

**Table 4:** Weight calculation results.

Overall Indicators	Secondary Indicators	Information Entropy (*d*)	Weights (W)
Medical Service Coverage Rate Index from Different Medical Orientations $(C_{i}$)		0.880714	18.54%
Comprehensive Accessibility Index for Multiple Transportation Modes $(A_{i}^{F}$)		0.977458	3.50%
Equity Index of Medical Facilities	Geographic factors $({FG}_{i}$)	0.810196	29.50%
	Population factors $({FP}_{i}$)	0.993955	0.94%
Supply and Demand Ratio Index of Medical Resources From the Perspective of Sudden Public Health Events $(V_{i}$)		0.694190	47.52%

**Table 5:** Grading of rationality assessment for medical resources spatial allocation.

Rationality Rating	Score	Percentage of Regional Area
Less rational	0–0.06	38.73%
Slightly rational	0.07–0.14	22.40%
Moderately rational	0.15–0.24	18.95%
Rational	0.25–0.36	14.24%
Very rational	0.37–0.71	5.68%

## Data Availability

Data are contained within the article.
